# METTL16 emerges as a pivotal epitranscriptomic regulator, linking RNA modification, tumor progression, and immune modulation

**DOI:** 10.3389/fimmu.2025.1706971

**Published:** 2025-11-14

**Authors:** Qiang Wang, Xiulin Jiang, Yixiao Yuan, Chunhong Li

**Affiliations:** 1 Department of Gastrointestinal Surgical Unit, Suining Central Hospital, Suining, Sichuan, China; 2 Department of Systems Biology, City of Hope Comprehensive Cancer Center Biomedical Research Center, Monrovia, CA, United States; 3 Department of Oncology, Suining Central Hospital, Suining, Sichuan, China

**Keywords:** METTL16, RNA methylation, cancer progression, TME, immunotherapy, biomarker, therapeutic target

## Abstract

N6-methyladenosine (m6A) modification has emerged as a critical epigenetic mechanism regulating gene expression in diverse physiological and pathological processes, including cancer. Methyltransferase-like 16 (METTL16), a recently identified m6A methyltransferase, has been shown to influence tumor progression through m6A-dependent regulation of key target genes. Accumulating evidence indicates that METTL16 exerts tumor-suppressive or tumor-promoting roles in a context-dependent manner, affecting cell proliferation, apoptosis, autophagy, and chemotherapeutic response across multiple cancer types such as bladder cancer, lung cancer, colorectal cancer, and acute myeloid leukemia. Mechanistically, METTL16 modifies the mRNA stability and translation of oncogenes or tumor suppressors via recognition of m6A sites, and its expression can be regulated by upstream factors including transcription factors and hypoxia-inducible signals. Recent evidence suggests that METTL16 also modulates the tumor microenvironment (TME), potentially affecting immune cell infiltration, immune checkpoint expression, and tumor immune evasion. Collectively, METTL16 emerges as a pivotal epitranscriptomic regulator linking RNA modification, tumor progression, and immune modulation, offering new avenues for precision oncology.

## 1 Introduction

RNA modifications, particularly m6A and other types of RNA modifications, represent a crucial layer of epitranscriptomic regulation, playing key roles in gene expression control, RNA stability, splicing, and translation efficiency (1). Recent studies have shown that aberrant RNA modifications are closely associated with the initiation, progression, and metastasis of various cancers (2). For instance, dysregulated expression of m6A methyltransferases can promote tumor cell proliferation, invasion, and drug resistance by modulating the expression of oncogenes or tumor suppressor genes (3). These findings suggest that RNA modifications not only constitute an important dimension of intracellular gene regulation but also hold potential diagnostic and therapeutic value in cancer.

METTL16 is an RNA methyltransferase belonging to the m6A methylation-associated protein family, yet its substrates and functional mechanisms differ substantially from those of the canonical METTL3/METTL14 complex (4). METTL16 primarily catalyzes m6A modification of specific U6 small nuclear RNAs (snRNAs) and mRNAs, contributing to RNA splicing, homeostasis maintenance, and metabolic regulation (5). For example, METTL16 can regulate the stability of MAT2A mRNA by recognizing specific RNA structural domains, thereby affecting intracellular S-adenosylmethionine (SAM) levels and indirectly modulating global RNA methylation (6). These functions of METTL16 underscore its critical roles in cell proliferation, stress responses, and metabolic homeostasis, suggesting a unique contribution to tumorigenesis and cancer progression.

Recent evidence indicates that METTL16 may also play a key role in the TME. Specifically, METTL16-mediated RNA modifications could influence the function and distribution of tumor-associated immune cells, such as tumor-infiltrating lymphocytes and macrophages, thereby contributing to immune evasion and therapy resistance (7). However, the precise mechanisms by which METTL16 regulates cancer immunity remain largely unclear, with most current studies focusing on *in vitro* systems or single tumor models. Systematic analyses and comprehensive reviews are still lacking. Therefore, this review aims to provide a thorough overview of the latest research on METTL16 in cancer development and tumor immune regulation, elucidate its potential molecular mechanisms and clinical implications, and highlight the prospective value of METTL16 as a diagnostic biomarker or immunotherapeutic target, offering a theoretical foundation for future basic and translational studies.

## 2 Biological functions of METTL16 in RNA regulation

METTL16 is a non-canonical RNA methyltransferase that primarily catalyzes m6Amodification on specific RNAs (5, 8). Compared with the canonical METTL3/METTL14 complex, METTL16 exhibits higher substrate specificity, typically recognizing RNA secondary structural features such as U6 snRNA, MAT2A mRNA, and certain non-coding RNAs (ncRNAs) (6). Beyond typical m6A modifications, studies suggest that METTL16 may also participate in atypical adenosine methylation, highlighting its diversity and specificity in RNA regulation.

In terms of RNA splicing, METTL16 can modify U6 snRNA or specific pre-mRNAs to regulate spliceosome assembly and splicing efficiency, thereby affecting mRNA maturation. Through this mechanism, METTL16 not only alters the expression levels of target genes but may also generate alternatively spliced protein isoforms with distinct functions, influencing tumor cell proliferation, survival, and stress responses (7). Regarding RNA stability, METTL16-mediated methylation can either enhance or reduce the stability of specific mRNAs. For example, modification of MAT2A mRNA maintains its stable expression, regulates intracellular SAM levels, and indirectly influences global RNA methylation (4). This homeostatic regulation impacts basic metabolism and may contribute to metabolic reprogramming in tumor cells, providing a growth advantage.

**Figure 1:**
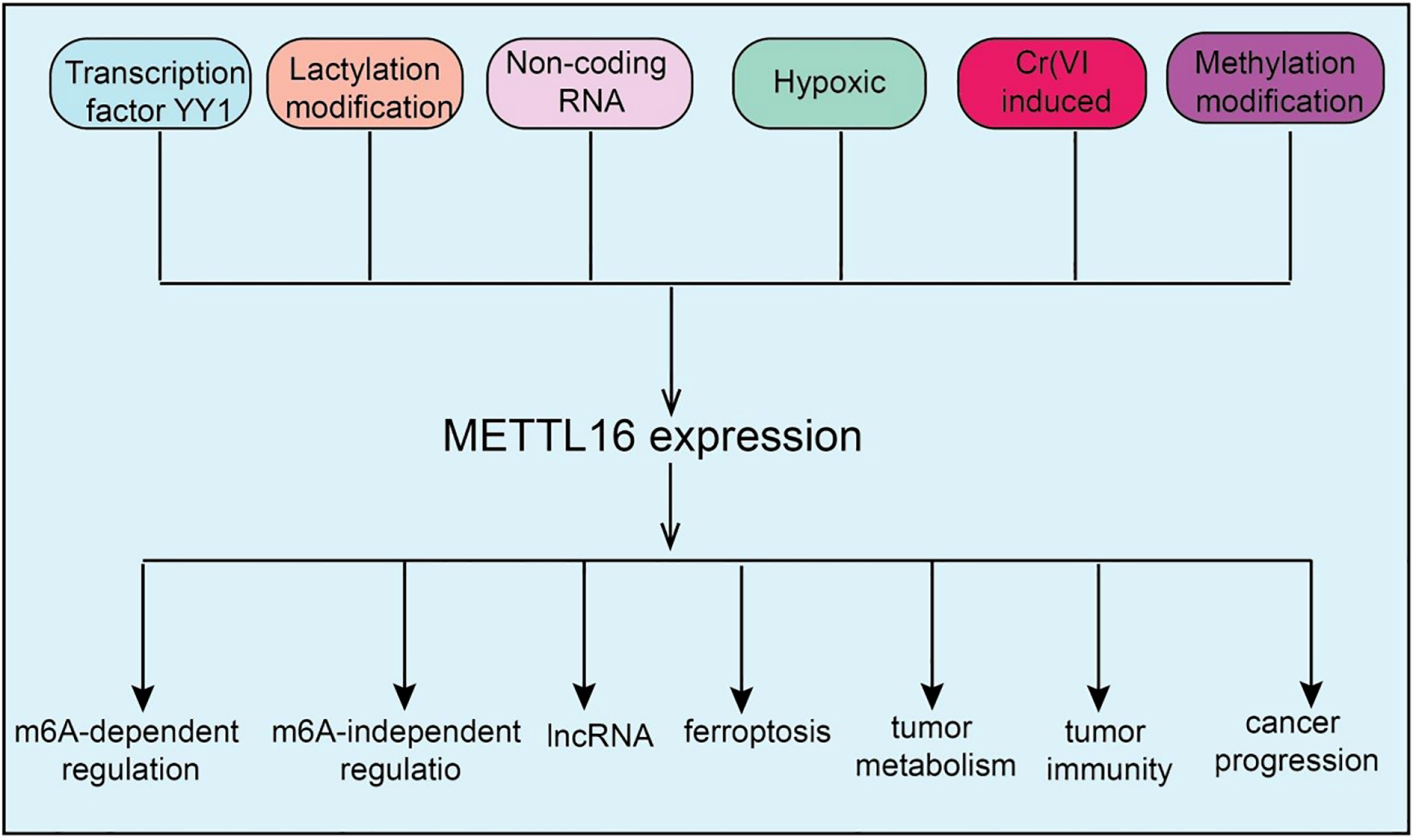
Upstream regulatory mechanisms of METTL16 and the downstream biological processes it participates in. The upstream regulation of METTL16 involves transcription factors, non-coding RNAs, and methylation modifications, while downstream, it exerts both m^6^A-dependent and m^6^A-independent functions to regulate biological processes such as ferroptosis, cancer metabolism, and tumor immunity.

In translational regulation, METTL16 affects mRNA nuclear export, ribosome recruitment, and translation efficiency, thereby modulating protein synthesis (9). This regulation influences not only normal cellular physiology but also the expression of key oncogenic or tumor-suppressive proteins in the tumor context, ultimately affecting cancer initiation, progression, and therapeutic response. Collectively, METTL16 orchestrates multi-layered RNA modification processes, including splicing, stability, and translation, playing a critical role in gene expression regulation and cellular homeostasis, which provides a molecular basis for its potential roles in tumor development and immune regulation(**Figure 1**).

## 3 Upstream regulatory mechanisms of METTL16 expression

As a key RNA methyltransferase, METTL16 expression is subject to multi-layered regulation at transcriptional, epitranscriptomic, and signaling levels. Current evidence indicates that METTL16 expression is finely tuned through transcriptional control, non-coding RNA-mediated regulation, post-translational modifications, and modulation by cellular stress and metabolic states. At the transcriptional level, the transcription factor YY1 can directly bind the METTL16 promoter, activating its transcription and thereby influencing downstream RNA methylation and associated biological processes (10). Multiple ncRNAs, including the lncRNA MALAT1 and U6 snRNA can modulate METTL16 post-transcriptionally by interacting with miRNAs or directly with METTL16 mRNA, affecting mRNA stability and translation efficiency (11). Post-translational modifications, such as protein methylation and lactylation, also play crucial roles in regulating METTL16, altering its stability, activity, and subcellular localization, and indirectly modulating its function (9). Notably, METTL16 expression can also be regulated by methylation-independent mechanisms, such as selective translational enhancement mediated by initiation factors eIF3A/B or eIF4E. Environmental factors, including hypoxia, can modulate METTL16 expression through HIF-1α/HIF-2α signaling pathways. Additionally, immunosuppressive factors and inhibitory signals within inflammation-related pathways, including NL and VXL, may participate in upstream regulation of METTL16, highlighting its potential role in the TME (12). Collectively, METTL16 expression is orchestrated by a complex network of upstream regulators, ensuring its dynamic expression and functional contribution to cell proliferation, metabolic regulation, and TME responses (**Figure 1**).

## 4 METTL16 in cancer progression

**Figure 2:**
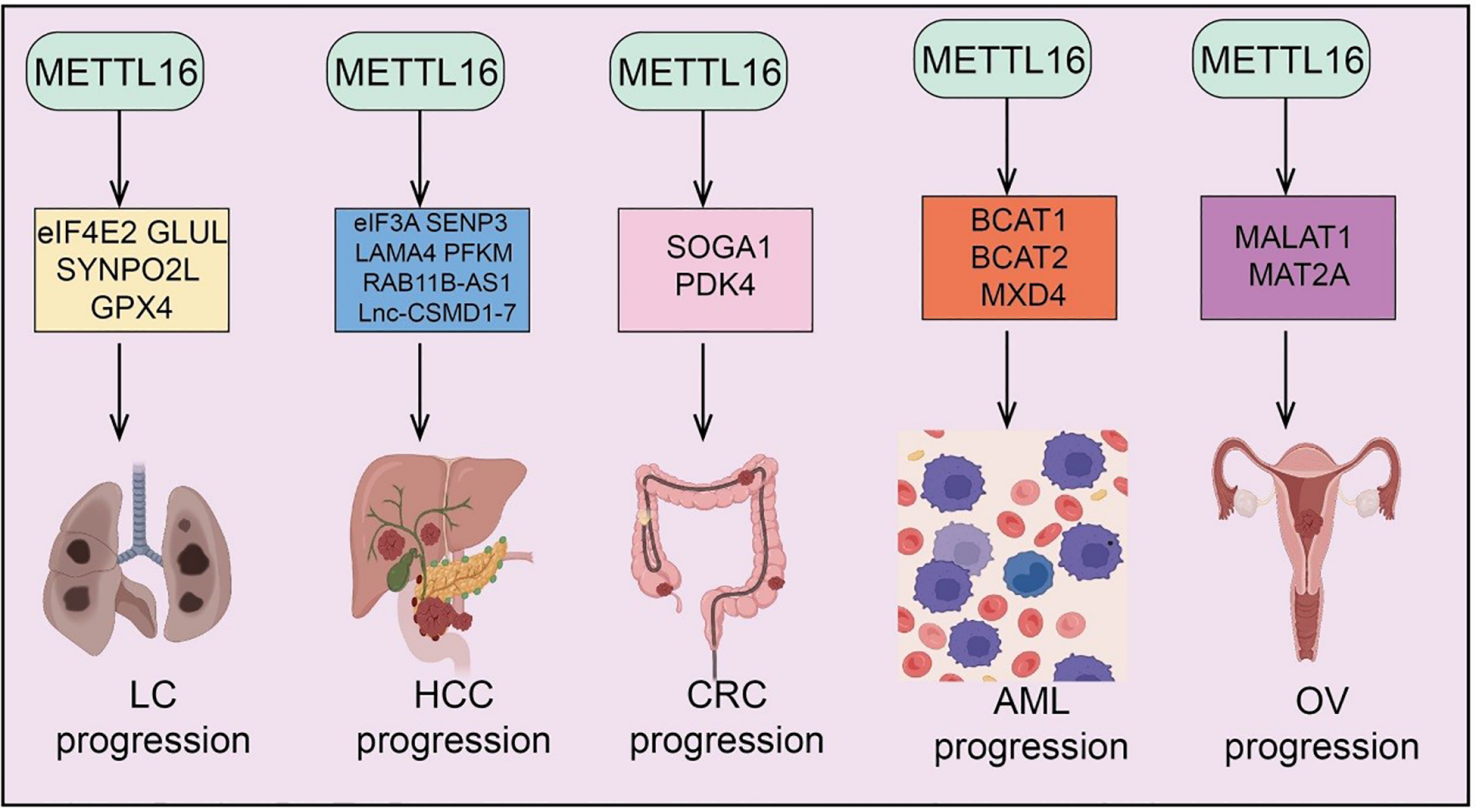
Target genes of METTL16 in LC, HCC, CRC, AML, and OV. METTL16 regulates the expression of various downstream target genes in an m^6^A modification–dependent manner, playing important roles and exerting key mechanisms in the progression of LC, HCC, CR), AML, and OV.

**Figure 3:**
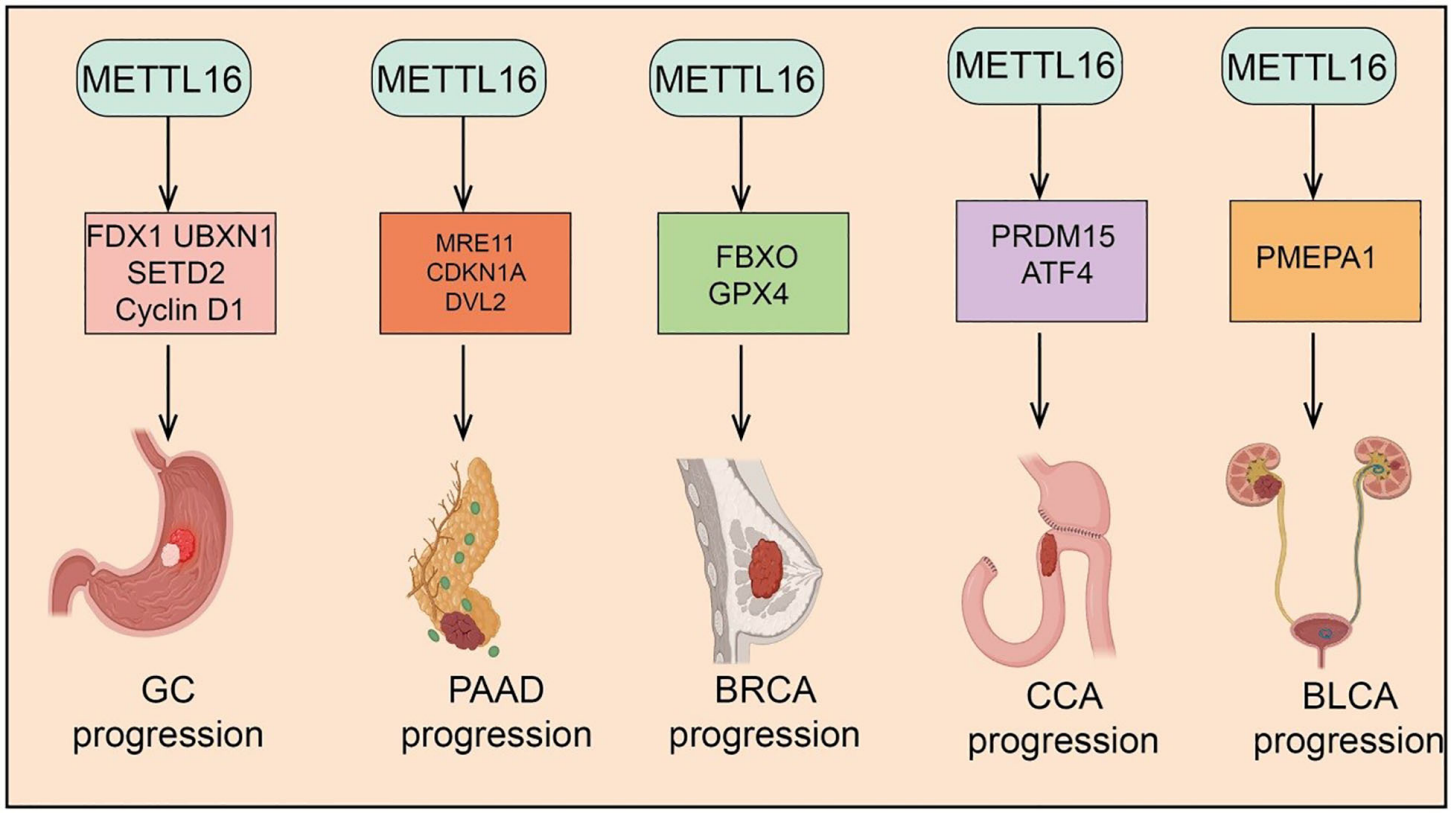
Target genes of METTL16 in GC, PAAD, CRC, CCA, and BLCA. METTL16 regulates the expression of various downstream target genes in an m^6^A modification–dependent manner, playing important roles and exerting key mechanisms in the progression of GC, PAAD, CRC, CCA, and BLCA. .

m6A modification, as a key epitranscriptomic regulator mechanism, plays a critical role in tumor initiation and progression (13). METTL16, an independent m6A methyltransferase, has recently been found to be widely involved in the regulation of multiple cancers, including lung cancer (LC), Hepatocellular carcinoma(HCC), Colorectal Cancer (CRC), Acute Myeloid Leukemia (AML), Ovarian Cancer (OV),Gastric Cancer (GC), Pancreatic Adenocarcinoma (PAAD), Breast Cancer, Cholangiocarcinoma (CCA) and Bladder Cancer (BLCA) (**Figure 2** and **Figure 3**). METTL16 exerts diverse functions across different tumor types by modulating cancer cell proliferation, migration, invasion, as well as the TME and immune responses (**Table 1**). The following sections will summarize the current research progress on METTL16 in these major cancers and discuss its potential clinical significance.

**Table 1:** Oncogenic and tumor-suppressive functions of METTL16 across cancers.

Cancer type	Role	Key targets	Function	Ref
LC	Oncogenic	GLUL (METTL16–MYC–GLUL–YTHDC1 axis), SYNPO2L (promotes CAF infiltration and EMT), GPX4 (ferroptosis inhibition, TKI resistance)	Promotes protein translation, metastasis, and drug resistance via both m6A-dependent and -independent mechanisms	(15–18)
HCC	Oncogenic	eIF3a, SENP3–LTF (ferroptosis resistance), LAMA4/COL4A1 (chemoresistance), PFKM (glycolysis reprogramming), Lnc-CSMD1-7 (tumor-suppressive lncRNA suppressed)	Regulates CSC self-renewal, chemoresistance, metabolism, and lncRNA-mediated tumor suppression	(12, 20–24)
CRC	Oncogenic	SOGA1–PDK4 (glycolysis), PLK1 (mitotic regulation)	Coordinates metabolic reprogramming and mitotic progression to promote tumorigenesis	(25, 26)
AML	Oncogenic	BCAT1/2–BCAA metabolism, MXD4–MYC pathway	Maintains leukemia stem cells, regulates metabolism and transcriptional programs	(28, 29)
OV	Tumor-suppressive	MALAT1–β-catenin, METTL16–MAT2A axis	Inhibits proliferation, migration, invasion, and tumor growth	(31, 32)
GC	Oncogenic	Cyclin D1 (mRNA stability), FDX1 (cuproptosis)	Promotes proliferation, cuproptosis, and histone-associated signaling	(9, 34, 35)
PAAD	Context-dependent	p21 (tumor-suppressive), DVL2–Wnt/β-catenin (oncogenic), MRE11 (DNA damage response)	Acts as tumor suppressor under normal/TME conditions; enhances survival or therapeutic sensitivity under DNA damage or treatment stress	(37–39)
BRCA	Oncogenic	FBXO5 (proliferation/EMT), GPX4 (ferroptosis inhibition)	Promotes proliferation, migration, EMT, and ferroptosis resistance; targeting METTL16 shows therapeutic potential in TNBC	(41–43)
CCA	Oncogenic	PRDM15, ATF4 (maintains mitochondrial homeostasis, inhibits ferroptosis)	Promotes proliferation, suppresses ferroptosis, and affects chemotherapy response	(10, 45)
BLCA	Tumor-suppressive	PMEPA1 (autophagy-mediated proliferation suppression)	High expression inhibits proliferation and enhances cisplatin sensitivity; HIF-2α can repress METTL16 transcription	(47)

### 4.1 LC

LC is a malignancy with high global incidence and mortality, and its complex molecular mechanisms and therapeutic challenges have driven the search for novel molecular targets (14). In this context, METTL16, a recently identified m6A methyltransferase, has attracted increasing attention for its potential roles and mechanisms in LC development. As a known nuclear single-component m6A methyltransferase, METTL16 localizes not only to the nucleus but is also present in the cytoplasm. Functional studies have revealed that loss of METTL16 significantly impairs protein synthesis, a function that is independent of its methyltransferase activity, suggesting an m6A-independent regulatory role. Mechanistically, METTL16 interacts with the translation repressor eIF4E2 (15). Under normal conditions, eIF4E2 inhibits translation by competing with eIF4E for the 5’ cap structure of mRNAs. METTL16 binding to eIF4E2 prevents its recruitment, thereby enhancing eIF4E recognition of the mRNA cap and promoting selective protein synthesis. Loss of METTL16 suppresses translation of key oncogenes, thereby markedly inhibiting LC initiation and progression (15). In metal-induced carcinogenesis, studies have shown that METTL16 expression is significantly upregulated in LC cells and mouse tissues exposed to hexavalent chromium [Cr(VI)] and plays a critical role in Cr(VI)-induced cell proliferation and tumor growth. High METTL16 expression has also been observed in various human cancer tissues (16). Mechanistically, METTL16 methylates specific stem-loop structures in its functional target GLUL in an m6A-dependent manner, enhancing the binding and splicing of the modified site by the m6A reader protein YTHDC1, thereby accelerating maturation of GLUL mRNA and upregulating GLUL expression. This promotes tumor development, highlighting the METTL16–MYC–GLUL–YTHDC1 axis as a key pathway in Cr(VI)-induced carcinogenesis and suggesting METTL16 as a potential therapeutic target for environmentally related cancers (16). In cancer metastasis, SYNPO2L has been identified as a core m6A-regulated gene whose high expression correlates with poor prognosis and metastasis. Mechanistic studies indicate that METTL16 regulates SYNPO2L stability via interaction with the m6A reader YTHDC1. SYNPO2L promotes COL10A1 secretion and enhances CAF infiltration, thereby inducing epithelial–mesenchymal transition (EMT) in tumor cells and facilitating distant metastasis (17). METTL16 has also been implicated in tyrosine kinase inhibitor (TKI) resistance in LC. In NSCLC tissues, METTL16 is significantly upregulated and associated with poor prognosis. Functional experiments show that METTL16 overexpression promotes NSCLC proliferation and resistance to AZD-9291, whereas METTL16 knockdown inhibits these phenotypes (17). Mechanistic studies combining bioinformatics and experimental validation identified GPX4 mRNA as a METTL16 substrate. In AZD-9291–resistant NSCLC cells (18), GPX4 expression is markedly increased, accompanied by inhibition of ferroptosis. METTL16 mediates m6A modification of GPX4, enhancing its stability and expression, thereby suppressing ferroptosis and promoting NSCLC proliferation and drug resistance. Targeting METTL16 restores ferroptosis activity and increases tumor sensitivity to AZD-9291 (18). In summary, METTL16 plays multifaceted roles in LC by regulating protein synthesis, oncogene translation, metastasis, and drug resistance through both m6A-dependent and -independent mechanisms. These findings establish METTL16 as a critical molecular regulator and a promising therapeutic target in LC.

### 4.2 HCC

Cancer stem cells (CSCs) are critical drivers of HCC initiation, progression, recurrence, and therapy resistance (19); however, the mechanisms underlying their self-renewal remain unclear. Studies have shown that METTL16 is highly expressed in liver CSCs, and its loss significantly reduces CSC frequency and inhibits their self-renewal both *in vitro* and *in vivo*. Further experiments demonstrate that METTL16 knockout markedly suppresses HCC initiation and progression while exerting only minimal effects on normal liver development, indicating a tumor-selective role. Mechanistically, METTL16 functions as a key regulator of ribosomal RNA maturation and mRNA translation, with eIF3a identified as a direct functional target in HCC (20). METTL16 is highly expressed in HCC, conferring resistance to ferroptosis and promoting cell viability and tumor progression. It cooperates with the m6A reader IGF2BP2 to stabilize SENP3 mRNA. SENP3, a deSUMOylase, inhibits proteasomal ubiquitination of lactoferrin (LTF), maintaining high LTF expression (21). Elevated LTF chelates free intracellular iron, reducing the labile iron pool and weakening ferroptosis. Functional studies confirm that SENP3 and LTF jointly mediate METTL16-driven HCC progression and ferroptosis resistance (21). METTL16 is also significantly upregulated in chemoresistant HCC tissues and cells. Knockdown of METTL16 suppresses cisplatin resistance, proliferation, invasion, and migration while promoting apoptosis. LAMA4 has been identified as a key METTL16 target. Through cooperation with IGF2BP2, METTL16 enhances m6A modification and stability of LAMA4 mRNA (22). LAMA4 interacts with COL4A1 to promote HCC progression and chemoresistance, partially offsetting the inhibitory effects of METTL16 knockdown. *In vivo*, METTL16 overexpression induces tumor growth, increases LAMA4 and COL4A1 expression, and enhances cisplatin resistance (22). In metabolic regulation, METTL16 overexpression in HCC correlates with poor prognosis. Knockdown significantly inhibits proliferation, invasion, spheroid formation, and glycolysis. Mechanistically, METTL16 stabilizes PFKM mRNA via m6A modification, dependent on IGF2BP3, maintaining PFKM expression and promoting glycolysis. Restoration of PFKM rescues the inhibitory effects of METTL16 loss (23). Upstream transcription factor POU3F2 activates METTL16 transcription. METTL16 also directly binds the lncRNA RAB11B-AS1, mediates its m6A modification, and decreases transcript stability, downregulating RAB11B-AS1. In contrast, RAB11B-AS1 is lowly expressed in HCC, and its downregulation correlates with poor prognosis (24). Functional experiments show RAB11B-AS1 inhibits HCC proliferation, migration, and invasion, promotes apoptosis, and suppresses tumor growth *in vivo*. Overexpression of RAB11B-AS1 can reverse the oncogenic effects of METTL16. Interestingly, in HCC, lncRNA Lnc-CSMD1–7 is downregulated and associated with poor prognosis. Functional assays demonstrate that Lnc-CSMD1–7 inhibits HCC cell migration and invasion and significantly suppresses lung metastasis *in vivo (*24). Mechanistically, Lnc-CSMD1–7 binds the splicing factor RBFOX2, regulating its alternative splicing activity in epithelial- versus mesenchymal-specific events, thereby exerting anti-metastatic effects. Further studies reveal that hypoxia upregulates METTL16 via HIF-1α. METTL16 directly binds Lnc-CSMD1–7 and reduces its RNA stability through m6A modification, leading to Lnc-CSMD1–7 downregulation and alleviating its inhibitory effects on HCC migration and metastasis (12). Clinically, high METTL16 and low Lnc-CSMD1–7 expression are closely associated with poor prognosis in HCC patients. Additionally, m6A-modified target gene ZNNT1 is highly expressed in HCC and correlates with unfavorable patient outcomes. In summary, METTL16 plays multifaceted roles in HCC by regulating CSC self-renewal, ferroptosis resistance, chemoresistance, metabolism, and lncRNA-mediated tumor suppression through both m6A-dependent and -independent mechanisms. These findings highlight METTL16 as a critical regulator and a potential therapeutic target in HCC.

### 4.3 CRC

CRC is one of the most common malignancies of the digestive system worldwide, with its development involving multiple genetic and epigenetic alterations and exhibiting highly variable prognoses (25). In recent years, RNA methylation, particularly m6A modification, has emerged as a critical regulator in CRC tumorigenesis, proliferation, and chemoresistance, drawing increasing attention. Among m6A regulators, METTL16 has been shown to play a significant role in CRC progression and metabolic reprogramming. In CRC glycolysis and tumor progression, METTL16 upregulates the expression and mRNA stability of its downstream target SOGA1 via m6A modification, a process dependent on the m6A reader protein IGF2BP1 (25). Functionally, SOGA1 promotes ubiquitination of the AMPK complex, thereby suppressing its expression and phosphorylation, which leads to upregulation of the key glycolytic regulator PDK4. This cascade enhances glycolytic capacity and the malignant phenotype of CRC cells. Furthermore, the transcription factor YY1 can inhibit METTL16 transcription by directly binding to its promoter. METTL16 expression positively correlates with SOGA1 and PDK4 levels and is closely associated with poor prognosis in CRC patients (25). Regarding chromosomal stability and mitotic regulation, loss of METTL16 or inhibition of its methyltransferase activity results in abnormal kinetochore–microtubule attachments during mitosis, causing delayed mitotic progression, chromosome lagging, mis-segregation, and chromosomal instability, thereby promoting CRC development. Mechanistically, METTL16 enhances SOGA1 expression in an m6A-dependent manner. SOGA1 can be phosphorylated by CDK1 and directly interacts with the polo-box domain of PLK1, facilitating PLK1 activation and driving mitotic progression (26). In summary, METTL16 promotes CRC tumorigenesis and progression by coordinating metabolic reprogramming and mitotic regulation via m6A-dependent modulation of SOGA1, highlighting its potential as a prognostic biomarker and therapeutic target in CRC.

### 4.4 AML

AML is a highly heterogeneous hematological malignancy characterized by rapid onset, aggressive progression, and poor prognosis (27). In recent years, RNA modifications, particularly m6A methylation, have been recognized as key regulators of AML initiation and progression. Among m6A regulators, the novel methyltransferase METTL16 has emerged as a focal point due to its ability to modulate gene expression and cellular fate. Studies have shown that METTL16 is aberrantly overexpressed in human AML cells, with enrichment in leukemia stem cells (LSCs) and leukemia-initiating cells (LICs). Functional experiments using CRISPR-Cas9 knockout demonstrate that METTL16 depletion significantly inhibits AML initiation, progression, and maintenance, markedly reducing LSC/LIC self-renewal while minimally affecting normal hematopoiesis (28). Mechanistically, METTL16 promotes the expression of BCAT1 and BCAT2 in an m6A-dependent manner, reprogramming branched-chain amino acid (BCAA) metabolism to provide metabolic support for leukemic cell survival and LSC maintenance. These findings reveal a critical role for the METTL16/m6A–BCAT1/2–BCAA axis in AML pathogenesis and stem cell regulation, highlighting METTL16’s role in epitranscriptomic and metabolic reprogramming as a driver of leukemogenesis and a potential therapeutic target (28). Similarly, METTL16 is highly expressed in primary AML cells, and its depletion or inhibition by small molecules significantly suppresses AML cell proliferation and induces apoptosis. Transcriptome analyses identify MXD4, a regulator of the MYC pathway, as a downstream target of METTL16 (29). Mechanistically, METTL16-mediated m6A modification reduces MXD4 mRNA stability, leading to decreased MXD4 protein levels, which indirectly activates the MYC–MAX complex and downstream target gene expression, thereby promoting AML cell proliferation and survival. Further studies show that MXD4 inhibition restores MYC target gene expression and reverses the growth-suppressive effects caused by METTL16 loss (29). In summary, METTL16 drives AML progression and leukemia stem cell maintenance through m6A-dependent modulation of metabolic and transcriptional pathways, establishing it as a key epitranscriptomic regulator and a promising therapeutic target in AML.

### 4.5 OV

OV is one of the deadliest malignancies of the female reproductive system, characterized by difficulty in early diagnosis and high recurrence rates (30). Recent studies indicate that RNA m6A modification plays a critical role in OV development, with the novel m6A methyltransferase METTL16 potentially regulating tumor proliferation, metastasis, and chemoresistance through modulation of key gene expression and signaling pathways. Studies have shown that METTL16 is significantly downregulated in epithelial ovarian cancer (EOC) tissues and cells, whereas its target lncRNA MALAT1 is highly expressed, exhibiting a negative correlation. Clinical analyses reveal that METTL16 downregulation is associated with poor prognosis in EOC patients. Functional experiments demonstrate that METTL16 markedly inhibits EOC cell proliferation, migration, and invasion, and suppresses tumor growth in subcutaneous xenograft mouse models. Mechanistically, METTL16 binds to MALAT1 and promotes its degradation, thereby reducing β-catenin protein expression and nuclear translocation, ultimately blocking downstream oncogenic signaling. This METTL16–MALAT1–β-catenin axis mediates the tumor-suppressive effects of METTL16 in OV (31). Similarly, METTL16 and MAT2A are highly expressed in OV tissues and cell lines (32). Treatment with 10 μg/mL of NCTD significantly inhibits ES2 and SKOV3 cell proliferation, induces apoptosis, suppresses migration and angiogenic activity, and downregulates related gene expression. Notably, METTL16 overexpression partially rescues the inhibitory effects of NCTD (32). In xenograft mouse models, NCTD administration significantly reduces tumor volume and downregulates METTL16, MAT2A, PP2A, and vascular endothelial growth factor (VEGF) expression. These findings indicate that NCTD exerts anti-OV effects by suppressing the METTL16–MAT2A signaling axis (32). In summary, METTL16 acts as a key regulator of OV progression through modulation of MALAT1 and β-catenin signaling, and targeting the METTL16–MAT2A axis may represent a promising therapeutic strategy for ovarian cancer.

### 4.6 GC

GC is a common malignancy of the digestive system worldwide, characterized by high invasiveness and poor prognosis (33). In recent years, RNA m6A modification has emerged as an important regulator in GC tumorigenesis, with the novel m6A methyltransferase METTL16 potentially modulating tumor proliferation, metastasis, and therapy resistance through regulation of key target genes and signaling pathways. Studies have shown that copper levels are significantly elevated in GC tissues, especially in malignant tumors, and METTL16 mediates cuproptosis by m6A modification of FDX1 mRNA. Mechanistic investigations further reveal that copper stress promotes lactylation of METTL16 at the K229 site, triggering cuproptosis, while SIRT2 inhibits this lactylation. Increased METTL16 lactylation significantly enhances the antitumor efficacy of the copper carrier drug elesclomol (9). Combination treatment with elesclomol and the SIRT2-specific inhibitor AGK2 induces cuproptosis in GC cells both *in vitro* and *in vivo (*9). Additionally, METTL16 cooperates with histone modifications via m6A to regulate GC tumorigenesis. METTL16 has been identified as a potential oncogene in GC, and its silencing suppresses malignant phenotypes while activating the NF-κB pathway. Multi-omics analyses reveal a strong positive correlation between METTL16 and UBXN1. Knockdown of METTL16 reduces m6A modification in the UBXN1 coding sequence and downregulates UBXN1 expression by promoting H3K36me3 modification at its promoter (34). METTL16 also modulates SETD2, a H3K36me3 methyltransferase, via m6A modification of its mRNA, indirectly influencing UBXN1 expression. Functionally, METTL16 is highly expressed in GC tissues and cells, and elevated levels are associated with poor patient prognosis. METTL16 promotes GC cell proliferation and tumor growth *in vivo*, whereas its downregulation inhibits proliferation by arresting the G1/S cell cycle transition (35). Mechanistically, Cyclin D1 is identified as a downstream effector of METTL16: METTL16 knockdown reduces global m6A levels, decreases Cyclin D1 mRNA stability, and suppresses Cyclin D1 expression, which is also affected by inhibition of methyltransferase activity. In summary, METTL16 promotes GC cell proliferation and tumor progression through m6A-mediated stabilization of Cyclin D1 and modulation of cuproptosis and histone-associated signaling, highlighting its potential as a therapeutic target in gastric cancer.

### 4.7 PAAD

PAAD is a highly aggressive malignancy of the digestive system, characterized by poor prognosis, late diagnosis, and strong resistance to chemotherapy (36). Recent studies have highlighted the critical role of RNA m6A modification in PAAD development, with the novel m6A methyltransferase METTL16 implicated in regulating key gene expression and signaling pathways, thereby influencing tumor proliferation, invasion, and therapeutic response. In PAAD, high METTL16 expression has been associated with increased sensitivity to PARP inhibitors (PARPi). Mechanistically, METTL16 interacts with MRE11 via RNA, inhibiting MRE11 exonuclease activity and blocking DNA end resection, independently of its methyltransferase activity (37). Under DNA damage conditions, ATM phosphorylates METTL16, inducing a conformational change that auto-inhibits its RNA binding and dissociates the METTL16–RNA–MRE11 complex, thus releasing MRE11 inhibition. Functionally, PAAD cells with high METTL16 expression exhibit enhanced sensitivity to PARPi monotherapy or combination therapy with gemcitabine (37). Conversely, other studies report downregulation of METTL16 in PAAD tissues and cells, identifying it as a protective factor in multivariate Cox regression analyses. Overexpression of METTL16 suppresses PAAD cell proliferation both *in vitro* and in xenograft models. Mechanistically, METTL16 regulates p21 via the METTL16–p21 axis (38); its downregulation reduces p21 expression, promoting cell proliferation. Changes in METTL16 expression also correlate with global m6A modification levels, indicating an m6A-dependent tumor-suppressive mechanism. Additional studies reveal that low METTL16 expression serves as a potential indicator of poor prognosis (38). Knockdown of METTL16 accelerates PAAD cell migration and invasion, whereas overexpression suppresses these malignant phenotypes. Mechanistically, METTL16 inhibits DVL2 translation via m6A modification, thereby regulating the Wnt/β-catenin signaling pathway; METTL16 downregulation leads to elevated DVL2 levels, promoting PAAD progression (39). In summary, METTL16 exhibits dual and context-dependent roles in PAAD: under normal physiological and TME conditions, it functions as a tumor suppressor by regulating cell cycle, signaling pathways, and immune responses; under DNA damage or specific treatment conditions, it can enhance cell survival or therapeutic sensitivity by modulating DNA repair and stress response pathways.

### 4.8 BC

BC is one of the most common malignancies in women, characterized by high heterogeneity and complex molecular mechanisms (40). Recent studies have revealed that RNA m6A modification contributes to breast cancer development, with the novel m6A methyltransferase METTL16 implicated in regulating key gene expression and signaling pathways, thereby affecting tumor cell proliferation, metastasis, and therapeutic sensitivity. In breast cancer, METTL16 is highly expressed in tumor tissues and cells, and its inhibition markedly suppresses BC cell proliferation, migration, and invasion. Mechanistically, METTL16 enhances FBXO5 mRNA stability via m6A modification, leading to elevated FBXO5 expression, which promotes cancer cell proliferation, migration, invasion, and EMT (41). Conversely, FBXO5 overexpression can rescue the tumor-suppressive effects of METTL16 knockdown, confirming the critical role of the METTL16–FBXO5 axis in breast cancer progression (41). Similarly, METTL16 is highly expressed in breast cancer patient tissues, and its knockdown not only inhibits BC cell proliferation *in vitro* and tumor growth *in vivo* but also significantly increases intracellular iron (Fe²^+^) and lipid reactive oxygen species (ROS) levels, indicating induction of ferroptosis. Mechanistically, METTL16 stabilizes GPX4 mRNA via m6A modification, increasing GPX4 expression and thereby inhibiting ferroptosis, which promotes BC cell survival and proliferation (42). Therapeutically, lipid nanoparticle (LNP)-based METTL16 targeting demonstrates antitumor efficacy in triple-negative breast cancer (TNBC). LNP/siMETTL16 efficiently silences METTL16 and markedly suppresses tumor cell activity. In subcutaneous tumor models, combination of LNP/siMETTL16 with LNP/mMUC1 achieves a 66.0% inhibition rate, showing synergistic antitumor effects. Intravenous administration of SORT-LNP/siMETTL16 combined with an mRNA tumor vaccine significantly mitigates TNBC lung metastasis, suggesting that organ-selective delivery combined with immunotherapy can enhance anti-metastatic treatment efficacy (43). In summary, METTL16 promotes breast cancer progression by regulating FBXO5-mediated proliferation and EMT as well as GPX4-mediated ferroptosis resistance, and targeting METTL16 via advanced nanoparticle-based delivery represents a promising therapeutic strategy for TNBC.

### 4.9 CCA

CCA is an aggressive malignancy originating from the bile duct epithelium, characterized by subtle early symptoms and poor prognosis (44). Recent studies have demonstrated that RNA m6A modification plays a critical regulatory role in CCA development and progression. The novel m6A methyltransferase METTL16 has been implicated in modulating key gene expression and signaling pathways, thereby influencing tumor proliferation, invasion, and chemotherapy response. In CCA, METTL16 is highly expressed in tumor tissues, and its knockdown significantly inhibits CCA cell proliferation and slows tumor progression (10). Mechanistically, PRDM15 has been identified as a critical downstream target of METTL16. METTL16 regulates PRDM15 protein expression via a YTHDF1-dependent translational mechanism, and restoration of PRDM15 expression can rescue the proliferation and colony formation defects caused by METTL16 depletion (10). Furthermore, the METTL16–PRDM15 axis also modulates FGFR4 expression, as PRDM15 binds to the FGFR4 promoter to regulate its transcription. Upstream regulation involves the histone acetyltransferase p300 cooperating with the transcription factor YY1 to activate METTL16 expression via H3K27 acetylation (45). Clinically, METTL16 overexpression in CCA tissues correlates with poor patient prognosis. Functionally, METTL16 maintains mitochondrial homeostasis—including mitochondrial structure, membrane potential, and energy production—thereby protecting CCA cells from ferroptosis while reducing Fe²^+^ metabolism and lipid peroxidation levels, promoting cell proliferation (45). Mechanistically, ATF4 has been identified as a novel METTL16 target: METTL16 mediates m6A modification of ATF4 mRNA, inhibiting its degradation and enhancing expression, which further suppresses ferroptosis (45). In summary, METTL16 drives CCA progression through m6A-dependent regulation of PRDM15 and ATF4, maintaining mitochondrial integrity and inhibiting ferroptosis, highlighting its potential as a prognostic biomarker and therapeutic target.

### 4.10 BLCA

BLCA is a common malignancy of the urinary system, characterized by high recurrence and invasiveness (46). Recent studies have revealed that RNA m6A modification plays a critical role in BLCA initiation and progression. The novel m6A methyltransferase METTL16 has emerged as a key regulator, modulating the expression of critical target genes and signaling pathways, thereby affecting tumor cell proliferation, chemotherapy sensitivity, and the TME. In BLCA, METTL16 functions as a tumor suppressor. Low METTL16 expression is associated with poor survival in patients, whereas high expression predicts favorable prognosis. Functional studies demonstrate that METTL16 inhibits bladder cancer cell proliferation both *in vitro* and *in vivo* and enhances cisplatin sensitivity. Mechanistically, METTL16 recognizes and binds the m6A site within the 3’-UTR of PMEPA1 mRNA, reducing its stability. This downregulation of PMEPA1 suppresses cell proliferation and enhances cisplatin sensitivity via PMEPA1-mediated autophagy pathways (47). Furthermore, HIF-2α promotes tumor progression by binding to the METTL16 promoter and repressing its transcription. Collectively, METTL16 regulates BLCA growth and chemotherapy response in an m6A-dependent manner through the HIF-2α–METTL16–PMEPA1–autophagy axis, providing novel mechanistic insights and potential therapeutic strategies for bladder cancer (47).

## 5 METTL16 and tumor immune regulation

### 5.1 Immune cells and METTL16 in cancer

**Figure 4:**
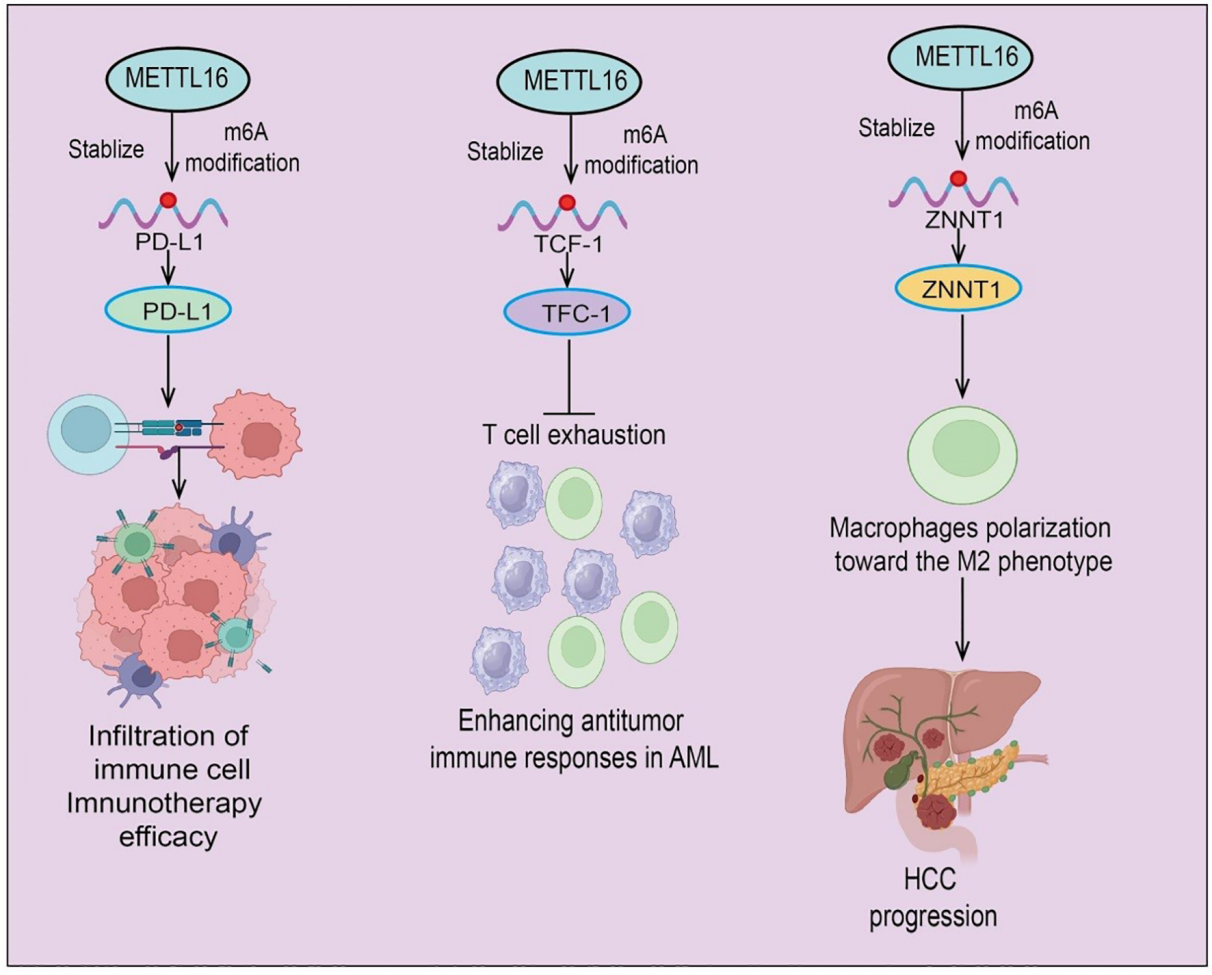
Schematic illustration of METTL16-mediated m6A modification in tumor immunity and progression. METTL16 acts as an epitranscriptomic regulator that stabilizes target transcripts through m6A modification. (Left) METTL16-mediated m6A modification of PD-L1 mRNA enhances its stability and expression, promoting immune cell infiltration and improving immunotherapy efficacy. (Middle) In acute myeloid leukemia (AML), METTL16 stabilizes TCF-1 mRNA via m6A modification, thereby preventing T cell exhaustion and enhancing antitumor immune responses. (Right) In hepatocellular carcinoma (HCC), METTL16-induced stabilization of ZNNT1 promotes macrophage polarization toward the immunosuppressive M2 phenotype, contributing to HCC progression.

Immune cells are central components of the host immune system, including T cells, B cells, natural killer (NK) cells, macrophages, and dendritic cells (DCs), which maintain immune homeostasis by recognizing and eliminating pathogens or tumor cells (48). Within the TIME, different immune cell types can exert antitumor functions or be hijacked by tumors to form an immunosuppressive milieu, thereby influencing tumor growth, metastasis, and therapeutic response (49). As a novel m6A methyltransferase, METTL16 may indirectly modulate immune cell functions and the TME by regulating the stability and translational efficiency of immune-related mRNAs. In PAAD, a complex multistep disease driven by mutations in KRAS, TP53, CDKN2A, and SMAD4, METTL16 is consistently downregulated across tissues and cell lines harboring these driver mutations. Its expression is closely associated with patient prognosis, highlighting its potential as a prognostic biomarker (7). Functional studies demonstrate that METTL16 exerts tumor-suppressive effects: upregulation inhibits tumor cell growth and invasion, while downregulation promotes tumor progression. Mechanistically, high METTL16 expression correlates positively with B cell and CD8^+^ T cell infiltration and modulates immune checkpoint molecules and cytokine expression. *In vitro*, METTL16 upregulation decreases PD-L1 expression, whereas its knockdown increases PD-L1 levels, indicating a regulatory role in immune evasion and antitumor immunity (7). These findings suggest that METTL16 functions not only as a potential prognostic biomarker but also as a tumor suppressor “writer” in PDA, influencing the TME and antitumor immune responses and highlighting its potential as an immunotherapeutic target (**Figure 4**).

In glioma, METTL16 knockdown inhibits tumor cell migration and invasion while inducing ferroptosis. Immune profiling shows altered proportions of CD8^+^ T cells, activated mast cells, and M2 macrophages in low-grade glioma compared with normal tissue. Notably, METTL16 expression negatively correlates with CD8^+^ T cell infiltration, whereas NFE2L2 expression positively correlates with M2 macrophages and immune checkpoints (50). In AML, METTL16 is upregulated, and high expression is significantly associated with poor prognosis. Functional studies reveal that METTL16 deletion enhances CAR-T cell persistence and cytotoxicity, promoting differentiation into TCF-1^+^ precursor exhausted T cells (TPEX) (51). Mechanistically, METTL16 mediates m6A modification of TCF-1 mRNA, reducing its stability and expression; TCF-1 loss promotes T cell exhaustion and inhibits self-renewal. METTL16 deletion thus enhances CAR-T cell durability and memory formation, strengthening antitumor immunity in AML, suggesting a potential strategy for immunotherapy targeting METTL16. In non-tumor contexts, such as diabetes mellitus (DM), lactate-driven tumor-associated Schwann cells (TASCs) contribute to immune suppression in diabetes-associated PDA. Single-cell RNA sequencing identifies a c1-Mettl16^+^Cd276^+^Nectin2^+^ TASC subpopulation that suppresses CD8^+^ T cell function and promotes PD-1-mediated immune tolerance. Mechanistically, lactate enters TASCs via MCT1/MCT4, interacts with METTL16, and induces K269 lactylation, enhancing m6A-dependent CTCF stability and activating transcription of immunosuppressive ligands (52). Targeting METTL16 restores immune surveillance and sensitizes tumors to PD-1 blockade. Retrospective clinical analyses further suggest that diabetic PDA patients may benefit from immunotherapy when treated with rosuvastatin. Moreover, METTL16 cooperates with METTL3 to mediate m6A modification of ZNNT1 mRNA, enhancing transcript stability and oncogenic expression. Mechanistically, ZNNT1 upregulates osteopontin (OPN) expression and secretion, recruiting macrophages and inducing M2 polarization (53). M2 macrophages secrete S100A9, which via the AGER/NF-κB signaling pathway further increases ZNNT1 expression in HCC cells, forming a ZNNT1–OPN–S100A9 positive feedback loop. This circuit not only promotes macrophage recruitment and M2 polarization but also enhances the malignant phenotype of HCC cells (53) (**Figure 4**). In summary, METTL16 plays a multifaceted role in regulating immune cell function, antitumor immunity, and the TME across multiple cancer types. Its modulation of m6A-dependent pathways affects immune infiltration, immune checkpoint expression, and tumor immune evasion, underscoring its potential as both a prognostic biomarker and a target for immunotherapy.

### 5.2 Immune checkpoints and METTL16

Immune checkpoints are critical regulatory molecules that modulate T cell activity, maintaining self-tolerance by preventing excessive immune responses. Within the TIME (54), tumor cells frequently upregulate immune checkpoint molecules such as PD-1, PD-L1, and CTLA-4, thereby suppressing T cell activation and antitumor immunity, which facilitates immune evasion. METTL16 may regulate the expression of immune checkpoint-related genes in an m6A-dependent manner, influencing tumor immune surveillance and the efficacy of immunotherapy. The advent of immune checkpoint inhibitors (ICIs) has dramatically transformed cancer treatment; however, response rates in CRC patients remain suboptimal. Studies indicate that METTL16 expression is reduced in CRC tissues and cell lines, whereas PD-L1 expression is elevated. Functionally, METTL16 promotes CRC cell proliferation, migration, and invasion, and enhances *in vivo* tumor growth (55). Mechanistically, METTL16 induces m6A modification on its own RNA, reducing transcript stability and thereby suppressing its expression. Overexpression of METTL16 in CRC cells decreases the proportion of PD-1^+^ T cells, and when combined with PD-1 blockade, METTL16 overexpression synergistically inhibits CRC tumor growth *in vivo*. Collectively, these findings suggest that METTL16 modulates immune checkpoint signaling through m6A-dependent mechanisms, highlighting its potential role in shaping antitumor immunity and enhancing the therapeutic efficacy of ICIs in CRC (55).

### 5.3 TME and METTL16

The TME is a complex ecosystem composed of tumor cells and surrounding non-tumor cells, immune cells, vasculature, stromal cells, and the extracellular matrix (56). The TME not only provides support for tumor growth, invasion, and metastasis but also influences tumor progression and therapeutic responses through mechanisms such as immunosuppression, angiogenesis, and regulation by signaling molecules. As a novel m6A methyltransferase, METTL16 may regulate the expression of tumor- and immune-related genes, thereby modulating immune cell infiltration, cytokine secretion, and immune checkpoint regulation within the TME, ultimately affecting tumor growth and antitumor immune responses. Research on METTL16 in the context of the TME remains limited, primarily due to its recent discovery and the fact that its biological functions and underlying regulatory mechanisms are not yet fully elucidated. Most current studies focus on METTL16’s role in tumor cell proliferation, autophagy, and chemosensitivity, whereas its involvement in immune cell differentiation, infiltration, and immunosuppressive or activating pathways remains underexplored. METTL16 may influence immune-related gene expression through complex m6A-dependent mechanisms, such as regulating the stability and translation efficiency of mRNAs encoding immune checkpoint molecules, cytokines, or antigen-presenting proteins, thereby indirectly modulating the immune composition and function of the TME. Potential molecular mechanisms include: Modulating tumor-derived immunoregulatory factors via m6A modification to influence the recruitment and activation of T cells, macrophages, and dendritic cells; Regulating key transcription factors or signaling pathways within immune cells-such as NF-κB, JAK/STAT, or HIF-related pathways-to alter the magnitude and type of immune responses; Collaborating with other m6A enzymes to form regulatory networks that impact tumor–immune cell interactions. Overall, the role of METTL16 in the TME remains a promising and largely unexplored area. A deeper understanding of its functions may reveal novel molecular targets for cancer immunotherapy.

Although METTL16 has emerged as an important m^6^A methyltransferase with diverse roles in RNA processing and cellular homeostasis, its involvement in tumor immunity remains poorly explored. One possible reason is that, compared with the well-characterized m^6^A “writer” components METTL3 and METTL14, the molecular mechanisms and regulatory targets of METTL16 are less well defined, making it more challenging to investigate its immunological functions. Additionally, most current studies on METTL16 have focused on its roles in RNA splicing, SAM homeostasis, and cancer cell–intrinsic signaling, rather than the TME. Another factor may be the lack of comprehensive datasets or functional studies directly linking METTL16 to immune cell regulation or immune evasion pathways. Looking forward, integrating multi-omics data (such as single-cell transcriptomics and epitranscriptomic profiling) with functional experiments will be crucial to elucidate the immunomodulatory functions of METTL16. Future research should explore whether METTL16 influences immune checkpoint expression, antigen presentation, or the recruitment and activation of immune cells within the TME. Such studies may uncover novel regulatory axes and therapeutic targets, broadening our understanding of RNA modifications in cancer immunity.

## 6 METTL16 as a potential biomarker and therapeutic target

Recent studies have demonstrated that aberrant expression of METTL16 is closely associated with clinical prognosis across multiple cancer types. In tumors such as hepatocellular carcinoma, gastric cancer, and breast cancer, high METTL16 expression is frequently correlated with enhanced tumor invasiveness, poor differentiation, and reduced overall survival (57). These findings suggest that METTL16 may serve as a potential prognostic biomarker, enabling assessment of disease progression risk in cancer patients. Moreover, METTL16 exhibits tissue- or cell-specific expression patterns in certain tumor types, and its stable expression profile may provide a basis for early diagnosis. In particular, combining METTL16 with conventional biomarkers could improve diagnostic sensitivity and specificity (58).

A recent study integrating multiple cancer databases systematically analyzed METTL16 expression across diverse human malignancies and its association with patient prognosis. METTL16 was found to be highly expressed in most tumor types and significantly correlated with poor clinical outcomes, with particularly pronounced relevance in CRC (58). Further *in vitro* experiments demonstrated that downregulation of METTL16 markedly inhibited CRC cell proliferation and migration. Collectively, these findings reveal the expression patterns and biological functions of METTL16 across cancers, providing evidence for its utility as a prognostic biomarker and highlighting its critical role in CRC. From a therapeutic perspective, the molecular functions and downstream pathways of METTL16 offer potential strategies for targeted intervention. Inhibition of METTL16’s methyltransferase activity can affect tumor cell RNA stability, splicing, and translation, thereby suppressing proliferation and invasion. Additionally, METTL16 may participate in tumor immune regulation by modulating immune cell function within the TME, providing a theoretical rationale for combination therapies with immune checkpoint inhibitors or other immunotherapies. Currently, small-molecule inhibitors and RNA interference strategies targeting METTL16 are showing promising results in preclinical studies, and may eventually serve as novel cancer treatment approaches. In summary, METTL16 demonstrates clear prognostic and diagnostic value, while its molecular mechanisms provide avenues for targeted intervention. These findings suggest that METTL16 has the potential to become a key target in precision oncology and cancer immunotherapy.

Recent studies have highlighted the roles of m6A methyltransferases in both cancer progression and immune regulation, yet different family members appear to exert distinct context-dependent effects. METTL16, as described in our review, demonstrates dual functionality across cancer types, acting as either a tumor suppressor or an oncogene depending on cellular context, TME, and specific downstream targets. Mechanistically, METTL16 regulates mRNA stability and translation via recognition of m6A sites on oncogenes or tumor suppressors, and its expression is modulated by upstream factors such as transcription factors and hypoxia-inducible signals. Furthermore, METTL16 influences the TME, potentially affecting immune cell infiltration, immune checkpoint expression, and tumor immune evasion. In comparison, METTL3, another core m6A writer, has been shown to play predominantly oncogenic roles in most cancers (59) and also critically regulates immune responses. For example, METTL3 overexpression can impair antiviral immunity and disrupt immune tolerance, thereby influencing susceptibility to viral infections and autoimmune diseases (60). Pan-cancer analyses indicate that METTL3 expression correlates with immune cell infiltration, tumor mutation burden, microsatellite instability, mismatch repair gene expression, and epithelial-mesenchymal transition, underscoring its broad impact on both tumor progression and immune modulation. Unlike METTL16, METTL3’s effects are more consistently pro-tumorigenic, although it similarly modulates the TME through RNA modification pathways. Taken together, these observations suggest that while both METTL16 and METTL3 mediate m6A-dependent regulation, METTL16 exhibits more context-specific and dualistic roles, whereas METTL3 functions predominantly as an oncogenic m6A writer with significant immune regulatory implications. This comparison underscores the importance of considering individual m6A regulators’ distinct mechanisms and context-dependent effects when evaluating their potential as therapeutic targets in cancer and immune modulation.

## 7 Challenges and future perspectives

Despite recent advances in METTL16 research, several challenges remain. First, most studies to date have focused on *in vitro* cell models or mechanistic analyses in specific cancer types, with limited validation in large-scale clinical samples. As a result, the expression patterns, functional roles, and clinical relevance of METTL16 across diverse cancers remain incompletely understood. This knowledge gap is particularly evident in the context of tumor immune regulation, where studies are scarce, likely due to METTL16’s complex substrate specificity, multifunctionality, and the intrinsic heterogeneity of the TME. The reported contradictory roles of METTL16 across different cancer types likely arise from several context-dependent factors. First, tumor heterogeneity plays a critical role: different cancers possess distinct genetic backgrounds, driver mutations, and signaling pathway activities, which can influence whether METTL16’s m6A-mediated regulation stabilizes oncogenes or tumor suppressors. For example, in LC, METTL16 may preferentially enhance the stability or translation of oncogenic transcripts, promoting proliferation and survival, whereas in bladder cancer, it may target tumor suppressor mRNAs for stabilization, thereby inhibiting tumor growth. Second, the TME contributes to these divergent effects; variations in hypoxia, immune cell infiltration, stromal components, and cytokine milieu can modulate METTL16 expression or its downstream targets, shaping its functional outcome. Third, isoform-specific functions or post-translational modifications of METTL16 may differentially regulate its substrate specificity or catalytic activity, leading to context-dependent oncogenic versus tumor-suppressive roles. Finally, upstream regulatory factors such as transcription factors, non-coding RNAs, or stress signals (e.g., hypoxia) can further dictate METTL16’s functional direction in a tissue-specific manner. Collectively, these factors highlight that METTL16’s role is highly context-dependent, and understanding its molecular interactions in each cancer type is essential for accurately evaluating its potential as a therapeutic target.

Moreover, METTL16 displays distinct target genes and regulatory mechanisms across cancer types. In HCC, METTL16 may influence ferroptosis and chemotherapy resistance via GPX4 or LAMA4/IGF2BP2 pathways. In AML, METTL16 predominantly regulates BCAT1/2 and MXD4 through m6A modifications (42), affecting branched-chain amino acid metabolism and MYC activity. In PAAD, METTL16 modulates DVL2 translation to regulate Wnt/β-catenin signaling, whereas in cholangiocarcinoma, it stabilizes ATF4 mRNA to suppress ferroptosis. These differences may reflect: (i) cancer-specific availability of RNA or protein substrates; (ii) tissue- or tumor-specific expression of m6A “readers” and “erasers”; and (iii) variations in TME and metabolic status, which influence METTL16 substrate selection and functional output.

At the basic research level, gene editing tools such as CRISPR-Cas9 provide powerful means to dissect METTL16 function, allowing direct evaluation of its effects on tumor proliferation, metastasis, and immune cell activity, thereby guiding mechanistic studies and therapeutic development. Whether METTL16 participates in liquid–liquid phase separation (LLPS) remains largely unknown. If METTL16 modulates the assembly of RNA or protein complexes via LLPS, this could provide mechanistic insight into its complex roles in RNA metabolism and immune regulation and suggest novel intervention strategies. From a translational perspective, targeting METTL16 in combination with immune checkpoint inhibitors (e.g., PD-1/PD-L1 blockade) holds potential. Modulating METTL16 activity or expression could simultaneously affect tumor proliferation and the immune microenvironment, enhancing immunotherapy efficacy. However, the safety, specificity, and applicability across different cancers remain to be rigorously validated.

Functionally, METTL16 exerts both m6A-dependent and -independent effects. Its m6A-dependent role mainly involves RNA modification, regulating target gene expression, stability, and splicing. The m6A-independent function may involve RNA structure recognition, protein interactions, or LLPS. Currently, it is unclear which of these functions predominates in tumorigenesis and immune modulation. Future studies should refine mechanistic insights and substrate specificity to delineate their relative contributions. In summary, although METTL16 research is still in its early stages, its potential roles in RNA metabolism, cancer progression, and tumor immunity warrant in-depth investigation. Future studies should focus on clinical sample validation, animal model development, LLPS-related mechanisms, cancer type-specific substrate identification, and targeted therapeutic strategies to clarify the diagnostic, prognostic, and immunotherapeutic potential of METTL16.

## 8 Conclusion

As a non-classical RNA methyltransferase, METTL16 exerts multifaceted roles in cancer development and tumor immune regulation. Through both m6A-dependent and -independent mechanisms, METTL16 modulates RNA splicing, stability, translation efficiency, and metabolic homeostasis, thereby influencing tumor cell proliferation, invasion, metastasis, and immune evasion. Moreover, its potential function within the TME provides a rationale for considering METTL16 as a combinatorial target in immunotherapy. Clinical studies and animal models indicate that aberrant METTL16 expression correlates with cancer prognosis, and its molecular functions offer opportunities for the development of targeted therapies and diagnostic biomarkers.

Future research should focus on several key directions: (i) validating METTL16 expression patterns and prognostic significance across diverse cancer types using large-scale clinical cohorts; (ii) employing gene-editing tools and animal models to elucidate its precise mechanisms in tumor progression and immune regulation; (iii) investigating whether METTL16 regulates RNA or protein complexes via novel mechanisms such as LLPS; and (iv) developing METTL16-targeted small-molecule inhibitors or combination immunotherapeutic strategies, with assessment of their safety and efficacy in cancer treatment. Collectively, these efforts are expected to further uncover the central role of METTL16 in cancer biology and provide novel strategies for precision oncology and immunotherapy.
